# Molecular Communication between Plants and Plant-Growth-Promoting Microorganisms for Stress Tolerance

**DOI:** 10.3390/microorganisms10061088

**Published:** 2022-05-25

**Authors:** Naeem Khan

**Affiliations:** Department of Agronomy, Institute of Food and Agricultural Sciences, University of Florida, Gainesville, FL 32611, USA; naeemkhan@ufl.edu or naeemkhan001@gmail.com

Plant-growth-promoting microorganisms are beneficial microbes that reside in the rhizosphere and roots of plants, direct their developmental process and induce systemic resistance. Plants select beneficial bacteria and help in their colonization through the secretion of root exudates. There is a complex interkingdom signaling between the host and microbes for mutual interaction, which is also influenced by environmental factors. An exchange of chemical signals was begun between microbes and plants to establish a positive or inhibitory interaction. Molecular communication was built up by encompassing chemical signals from microbes to microbes, plants to microbes or microbes to plants, which leads to a cellular response and altered gene expression. Microorganisms are also known for their role in altering the metabolomics expression of host plants and inducing their systematic resistance by increasing the expression of stress-responsive secondary metabolites. Root exudates contain low-molecular-weight primary metabolites, such as carbohydrates, amino acids, organic acids, and high-molecular-weight secondary metabolites, e.g., alkaloids, terpenoids, phenolics, mucilage, proteins, and volatile organic compounds (VOCs). Many compounds of root exudates act as general chemoattractants, able to attract beneficial microbes and repel pathogens. Plant-growth-promoting microorganisms can enhance crops’ tolerance to various environmental stresses by improving the level of cellular metabolites, which suggests a microorganisms’ novel role of interacting with the plant metabolome, as well as influencing the plant microbiome. The above-mentioned examples of signaling molecules, along with thousands of others, mediate a complex network of signals in the rhizosphere, which helps plants to flourish and withstand abiotic stresses.

Abiotic stresses such as drought, salt, heavy metals, and high and low temperature are the common environmental constraints on crop production and future food security around the world. Among the abiotic stresses, soil salinity has more adverse effects on plants due to its severe effects on root growth. It is mainly caused by groundwater irrigation, where it accumulates in the rhizosphere, decreasing agricultural production. It is estimated that about 50% of the world’s arable land will be salt-affected by 2050, and the % increase is expected to be more due to frequent global climate change, thus endangering plant growth and productivity [1]. At present, more than 20% of cultivated lands are facing the problem of soil salinization. This results in a high accumulation of Na^+^ and Cl^−^ in the rhizosphere region of plants, which significantly reduces the root’s ability to absorb more water and other mineral ions required for normal plant growth, thereby inhibiting the overall growth of plants [2,3].

Plant roots tend to establish a symbiotic relationship with many soil microorganisms to deal with the negative impacts of salt stress. Arbuscular mycorrhizal fungi (AMF) are obligate symbiotic fungi that help plants to flourish well under salinity stress and can establish a symbiotic relationship with more than 80% of plants on the land. These fungi help plants to absorb more water and minerals under abiotic stresses, thus fulfilling their need to grow well under stresses. Current studies show that AMF can lessen the negative effects of salt stress and promote plant growth in such environmental conditions [4,5]. This increase in plant tolerance to salt stress by AMF is due to their ability to enhance water and nutrient absorption, maintain a better osmotic state, and increase the photosynthesis rate. Microbial inoculation can show plant reactions to salt stress in the presence of a plant-beneficial microorganism, and the results of these studies suggest that osmolyte accumulation and the anti-oxidative enzymatic defense system (e.g., catalase, POD, SOD, etc.) detoxify and neutralize the salinity-induced excess of reactive oxygen species (ROS). Recently, Sharma et al. [6] showed that the supplementation of salt-tolerant PGPR strain ASN-1 (*Bacillus xiamenensis*) and sodium nitroprusside (SNP) as a nitric oxide (NO) donor efficiently protected the sugarcane against the adverse effects of salt stress by managing the physiological, biochemical, and photosynthetic traits, and through the accretion of compatible solutes and antioxidant enzyme activities. The results showed that the combined application of both these bacterial species was more effective against high levels of soil salinity than their individual application. They also noted that the combined application of both these strains also effectively improved the plant growth under optimal growth conditions. Therefore, in the era of climatic changes, the application of PGPR isolate ASN-1 and SNP could be a promising approach for salt-stress management. The current data can be used to drive research efforts, aiming to increase abiotic stress tolerance and yield sustainability in agriculturally important crops.

Similarly, Wang et al. [7] studied the key NaCl stress response pathways and genes mediated by *Rhizophagus irregularis*. The results of this study are important to understand the insights into the salt tolerance mechanisms induced by *R. irregularis*. They reported that the application of *R. irregularis* significantly improved the growth of *Casuarina glauca*, regulated ion balance, and modulated the activity of antioxidant enzymes. They also carried out a transcriptome analysis of roots, which revealed that 1827 differentially expressed genes (DEGs) were affected by both *R. irregularis* inoculation and NaCl stress. The GO and KEGG enrichment analysis of these DEGs revealed that *R. irregularis* inoculation enhanced the NaCl tolerance of *Casuarina glauca* roots by regulating transcription factors, antioxidant enzymes activity, ion transport, and carbohydrate metabolism. The mycorrhizal *C. glauca* improved the injuries caused due by salt stress through *HAK5*, *CPER*, *KAT3*, *SKOR*, *PIP1-2*, *PER64*, *NAC43*, *GLP10*, *MYB46*, *WRKY1*, and *WRKY19*, which provide molecular evidence that *R. irregularis* inoculation can assist *C. glauca* in releasing salt stress. This supports the visual expression of the salt tolerance mechanism induced by AMF and sets a foundation for the future use of AMF to improve plant tolerance to salt stress.

The beneficial soil microbes also play an important role in the phytoremediation of heavy metals from the soil, and the use of metal-resistant bacteria is an environmentally friendly and cost-effective technique for wastewater treatment. Soil bacteria develop a continual contact with the pollutants present in the soil or in the polluted water used for irrigation [8,9]. Recently, Ali et al. [10] indicated the bacterial species that can be used in the remediation of heavy metals from the rhizosphere. For the first time, they used a consortium of two different bacteria (i.e., *Bacillus subtilis* and *Staphylococcus aureus*) to reduce the chromium (Cr) from the rhizosphere of castor beans. The used bacterial species also possess antiviral and antifungal activities, and thus protect the plants from their deleterious effects. Earlier research on bacterial antagonists shows that *Bacillus amyloliquefaciens* VB7 has a broad spectrum of action against *Sclerotinia sclerotiorum*, and groundnut bud necrosis, infecting cotton and tomato, respectively. The genome annotation of *B. amyloliquefaciens* VB7 was discovered to be *Bacillus. velezensis* VB7 (CP047587). The assembled genome was used to detect the presence of NRPS gene clusters encoding surfactin, butirosin A/butirosin B, fengycin, difficidin, bacillibactin, bacilysin and mersacidin the Ripp lanthipeptide, which are accountable for antifungal and antiviral action through wet lab confirmation. Thus, the present investigation has provided new means of exploring *B. velezensis* VB7 at a commercial scale to manage tomato spotted wilt virus, groundnut bud necrosis virus, tobacco streak virus, *S*. *sclerotiorum*, and *Fusarium oxysporum* f.sp. *cubense* (*Foc*) causing panama wilt of banana [11].

## Data Availability

All the data generated or analyzed during this study are included in this published editorial.
